# Antiosteoporotic effect of *Petroselinum crispum*, *Ocimum basilicum* and *Cichorium intybus L.* in glucocorticoid-induced osteoporosis in rats

**DOI:** 10.1186/s12906-016-1140-y

**Published:** 2016-06-02

**Authors:** Walaa G. Hozayen, Mohamed A. El-Desouky, Hanan A. Soliman, Rasha R. Ahmed, Amal K. Khaliefa

**Affiliations:** Chemistry Department, Faculty of Science, Beni-Suef University, Beni-Suef, Egypt; Biotechnology Department, Faculty of Postgraduate Studies for Advanced Sciences, Beni-Suef University, Beni-Suef, Egypt; Chemistry Department, Faculty of Science, Cairo University, Giza, Egypt; Zoology Department, Faculty of Science, Beni-Suef University, Beni-Suef, Egypt

**Keywords:** Dexamethasone, Osteoporosis, Bone biomarkers, Histopathology and oxidative stress

## Abstract

**Background:**

Glucocorticoid-induced osteoporosis (GIO) is one of the serious side effects which have become the most common secondary osteoporosis. The purpose of this study is to evaluate the effect of aqueous extract of parsley, basil and chicory on glucocorticoid-induced osteoporosis in rats.

**Methods:**

Fifty Female rats were divided into five groups and treated for 8 weeks as follow: group 1 served as control; group (2) subcutaneously injected with 0.1 mg/kg b. wt. dexamethasone dissolved in saline; group 3 received similar dose of dexamethasone together with aqueous parsley extract in a dose of 2 g/kg b. wt.; group 4 received similar dose of dexamethasone together with 400 mg/kg b. wt. aqueous basil extract and group 5 received similar dose of dexamethasone together with 100 mg/kg b. wt. aqueous chicory extract.

**Results:**

The dexamethasone group showed a significant decrease in serum E2, Ca, P levels and significant decrease in total BMD, BMC and a significant increase in serum PTH, ALP and ACP. Bone TBARs was significantly increased while GSH, antioxidant enzymes were significantly decreased. These changes were attenuated by parsley, basil and chicory extracts in the group 3, 4 and 5 respectively.

**Conclusion:**

Aqueous extracts of parsley, basil and chicory showed bone protection against glucocorticoid-induced in rats. From our results, we concluded that chicory has a potent protective effect more than parsley and basil due to containing flavonoids and inulin.

## Background

Glucocorticoids (GCs) are widely used to treat various inflammatory, immunologic and allergic disorders that cause rheumatic, respiratory, bowel, hepatic, neurological, renal and skin diseases [1]. Osteoporosis is one of the main complications of glucocorticoid application [2]. GC therapy suppresses osteoblast function, increases bone resorption, decreases calcium gut absorption, and suppresses endogenous gonadal steroids, all of which lead to increase bone loss [3].

Aqueous parsley extract has been used for the treatment of diseases or conditions characterized by increased bone resorption [4]. Parsley contains both calcium and vitamin c, as well as ergosterol, a precursor of vitamin D, which helps the body to absorb and utilize calcium [5]. Phytochemical screening of parsley has revealed the presence of flavonoids (apiin, luteolin, and apigenin-glycosides), the methanolic extract from the aerial parts of parsley showed potent estrogenic activity which is equal to that of isoflavone glycosides from soybean [6, 7].

The profound medical effects of basil may be attributed to its antioxidant power due to flavonoids and polyphenols content [8]. Flavonoids also called phytoestrogens because of their weak estrogenic activity with a chemical structure similar to 17β-estradiol, the most potent, naturally occurring estrogen as isoflavones bind to estrogen receptors, affecting estrogen-regulated processes [9, 10]. Phytoestrogens prevent bone resorption, and maintain or increase bone density and may inhibit osteoporosis to some degree in postmenopausal women, owing to their estrogenic activity because they are unlikely to cause the undesirable effects associated with steroid hormones [11, 12].

All parts of chicory plant possess medicinal importance as alkaloids, inulin, sesquiterpene lactones, coumarins, chlorophyll pigments, unsaturated sterols, flavonoids, saponins and tannins [13]. Dietary supplementation with inulin-type fructans enhances the uptake of Ca, improves bone mineral content (BMC) in growing rats and alleviates the reduction in bone mineral content and bone mineral density (BMD) which follows ovariectomy or gastrectomy in rats [14].

The aim of this study was to investigate the anti-osteoporotic action of aqueous extract of *Petroselinum crispum*, *Ocimum basilicum* and *Cichorium intybus L*. in rats administered dexamethasone and the antioxidant capability of these extracts.

## Methods

This work was conducted in the Chemistry Department, College of Science, Beni-Suef University.

Female albino rats (Rattus norvegicus) weighing about 120–150 gm. were used for the study and were kept in animal house at 26 ± 2 ° C with relative humidity 44 to 56 % along with light and dark cycles of 12 h, respectively. Animals were provided with standard diet and water ad libitum. All animal procedures were conducted in accordance with the standards set forth in the guidelines for the care and use of experimental animals by the Committee for the Purpose of Control and Supervision of Experiments on Animals (CPCSEA) and the National Institutes of Health (NIH). The study protocol was approved by the Animal Ethics Committee of the Zoology Department in the College of Science at Beni-Suef University.

### Chemicals

Dexamethasone [(Fortecortine® 8 mg – Mono ampoule) manufactured by Sigma – Tec Pharmaeutical industries – Egypt – S. A. E. under Licence of: Merck, Darmstadt, Germany]. Standard flavonoids (Luteo.6-arabinose8-glucose, Luteo.6-glucose8-arabinose, Apig.6-arabinose8-galactose, Apig.6-rhamnose8-glucose, Apig.6-glucose8-rhamnose, Naringin, Rutin, Hespirdin, Rosmarinic acid, Apig.7-o-neohespiroside, Apigenin-7-glucose, Quercetrin, Quercitin, Naringenin, Hespirtin, Kampferol, Apigenin) and saccharides (Glucuronic acid, Sucrose, Xylose, Rhaminose, Mannose, Arabinose, Manitol, Stachylose, Inulin, Fructose, Glucose) were purchased from Sigma Aldrich. Kit No. ES180S-100 purchased from Calbiotech. U.S. for E2 determination, kit No. MBS702121 purchased from My Biosource. U. S. A. for PTH determination, a commercial assay kit provided from Spinreact, Spain for Ca, P determination. Kit purchased from BioSystems Company, Spain for determination activity of alkaline phosophatase. Kit purchased from BIO Diagnostic Company, Egypt for determination activity of ACP and oxidative stress markers.

### Plant materials

Parsley plant (*P. crispum*), Basil plant (*O. basilicum*), Chicory plant (*C. intybus L.*) leaves were collected from herbal medicine market (Cairo, Egypt) and identified by an ecologist in plant department, Faculty of Science, Beni-Suef University. A voucher specimen was deposited in the herbarium of the Botany Department, College of Science, Beni-Suef University, Egypt.

### Preparation of aqueous parsley extract

The air dried parsley leaves (100 gm.) were extracted by adding 1000 ml of distilled water and boiled for 30 min. The extract was then filtered, and then filterate was evaporated, using rotary evaporator under reduced pressure to dryness (at 45 °C). The extract was dissolved in distilled water before the administration to rats [15].

### Preparation of aqueous basil extract

The ground powder of dried basil leaves (300 gm.) was infused for 30 min in 200 ml of distilled water at 100 °C followed by filteration. The solution obtained was concentrated rotary evaporator under a vacuum at 65 °C. The resulting crude extract was suspended in 30 ml sterile distilled water and aliquots were stored at −20 °C till use [8].

### Preparation of aqueous chicory extract

The powdered chicory leaves were added to the already boiling distilled water and infused for 15 min. Then, the infusion (2 % w/v) was filtered and the filtrate was freshly used [16].

### Identification of flavonoids in extracts by HPLC analysis

The flavonoid compounds of the samples were extracted according to the method described [17]. Three milliliters were collected in a vial for subsequent HPLC separation. HPLC instrument (Hewlett Packard, series 1050, country) equipped with stainless-steel column (Zorbax ODS 5 μ m 4.6 × 250 mm). Injection volume was 75 μl carried out with auto-sampling injector. The column temperature was maintained at 35 °C. Gradient separation was carried out with methanol and acetonitrile as a mobile phase at flow rate 1.0 ml/min. Elutes were monitored using UV detector set at 330 nm for flavonoid. Chromatographic peaks were identified by comparing the retention times with the respective retention times of known standard reference material.

### Identification of saccharides in extracts by HPLC analysis

Sugar profiles were determined by the method described [18] high performance with modification that liquid chromatography coupled to a refraction index detector (HPLC-RI). Soluble sugar determined at 80 °C. The HPLC system was equipped with a Hewlett Packard 1050 HP1047A RI detector and with HPLC instrument (Hewlett Packard, series 1050, country) equipped with stainless-steel column (Zorbax ODS 5 μ m 4.6 × 250 mm). The mobile phase was isocratic elution system was used by deionized water at a flow rate of 1 ml/min. Sugar identification was made by comparing the relative retention times of samples peaks with standards.

### Animal groups

For the achievement of the objectives of this study, 50 female albino rats were randomly divided equally into the following 5 groups:

- Group 1: served as normal control.
- Group 2: was given dexamethasone subcutaneously at 0.1 mg/kg b. wt./day dissolved in saline [ 19] and is considered as a control for groups 3, 4 & 5.
- Group 3: received 0.1 mg/kg. b. wt. of dexamethasone together with 2 g/kg b. wt. of *Petroselinum crispum* leaves aqueous extract [15].
- Group 4: received 0.1 mg/kg. b. wt. of dexamethasone together with 400 mg/kg b. wt. of *Ocimum basilicum* leaves aqueous extract [20].
- Group 5: received 0.1 mg/kg. b. wt. of dexamethasone together with 100 mg/kg b. wt. of *Cichorium intybus L.* leaves aqueous extract [21].

All these groups were treated for three times per week for 8 consecutive weeks and the treatments with parsley, basil and chicory were performed orally between 7.00 and 9.00 a.m.

### Biochemical assay

At the end of the experimental period (8 weeks), rats were sacrificed under diethyl ether anesthesia. Blood samples were collected from each rat, allowed to coagulate at room temperature then centrifuged at 3000 r.p.m. for 20 min. The clear, non hemolysed supernatant sera were quickly removed and kept at −20 °C till examined. For bone samples the left femurs were immediately removed, washed using chilled saline solution, weighed and minced in ice-cold 0.9 % saline solution using homogenizer. The homogenates were centrifuged, and the resultant supernatants were frozen at −20 °C [22].

The concentration of serum estradiol (E2) was determined by enzyme linked immunosorbent assay (ELISA) procedure [23]. The concentration of serum parathyroid hormone (PTH) was determined by ELISA procedure [24]. Serum and bone calcium and phosphorus concentrations were assayed according to the method of [25, 26] respectively. The activity of alkaline phosphatase in serum and bone was determined kinetically [27]. Acid phosphatase activity in serum and bone was determined colorimetrically [28].

### Bone mineral density and bone mineral content assay

The right femur of each animal was dissected and carefully cleaned for measuring bone mineral density (BMD) and bone mineral content (BMC) by dual energy x-ray absorptiometry (DEXA) using Norland XR 46, version 3.9.6/2.3.1 instrument equipped with dedicated software for small animal measurements in bone mineral density unit, Medical Service Unit, National research Center, Dokki, Egypt., this technique provides an software measure of right femur proximal, middle, distal and total areas.

### Bone oxidative stress and antioxidant enzymes assay

The left femur of each animal was homogenized in cold 0.9 % NaCl to make up to 10 % homogenate (w/v). The homogenates were centrifuged, and the clear supernatants were used for estimation of malondialdehyde (MDA) [29], glutathione (GSH) [30], glutathione-S-transferase (GST) [31], glutathione peroxidase (GPx) [32], glutathione reductase (GR) [33] and catalase [34] levels.

### Histopathological examination of bone

Right femur specimens were fixed in 10 % neutral buffered formalin for 24 h, decalcified in 10 % EDTA solution (pH = 7.4) and then processed till embedding in paraffin. Thin paraffin sections (4 μm) were stained with H&E [35].

### Statistical analysis

The data were analyzed using the one-way analysis of variance (ANOVA) [36] followed by LSD test to compare various groups with each other. Results were expressed as mean ± standard deviation (SD) and values of *P* > 0.05 were considered non-significantly different, while those of *P* < 0.05, *P* < 0.01 and *P* < 0.001 were considered significant, highly and very highly significant, respectively.

## Results

**Fig. 1:**
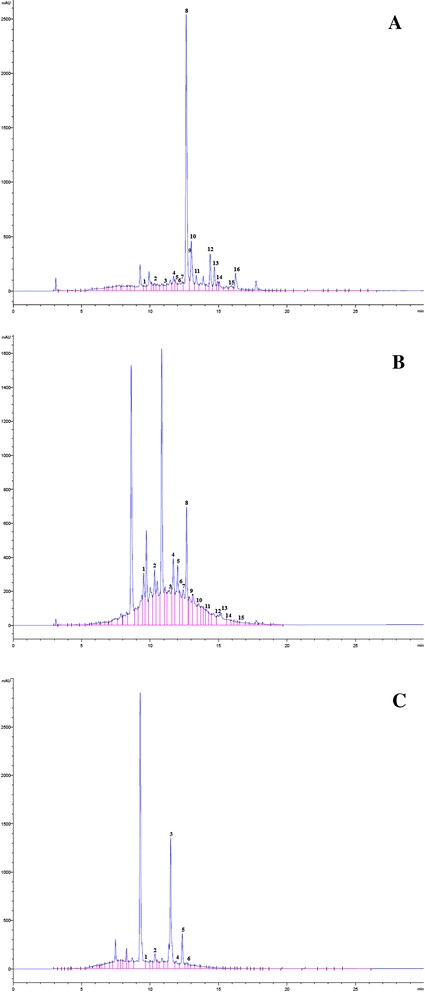
HPLC chromatogram (250-350 nm) of the flavonoid compounds of: **a**: Petroselinum crispum leaf. The peaks were identified (1) luteo.6-arabinose8-glucose, (2) luteo.6-glucose8-arabinose, (3) apig.6-rhaminose8-glucose, (4) apig.6-glucose8-rhaminose, (5) naringin, (6) rutin, (7) hespirdin, (8) rosmarinic, (9) apig.7-o-neohespiroside, (10) apiginin-7-glucose, (11) quercetrin, (12) quercitin, (13) naringenin, (14) hespirtin, (15) kampferol, (16) apigenin. **b**: Ocimum basilicum leaf. The peaks were identified (1) luteo.6-arabinose8-glucose, (2) luteo.6-glucose8-arabinose, (3) apig.6-rhaminose8-galactose, (4) apig.6-rhaminose8-glucose, (5) apig.6-glucose8-rhaminose, (6) rutin, (7) hespirdin, (8) rosmarinic, (9) apig.7-o-neohespiroside, (10) apiginin-7-glucose, (11) quercitin, (12) naringenin, (13) hespirtin, (14) kampferol, (15) apigenin. **c**: Cichorium intybus L. leaf. The peaks were identified (1) luteo.6-arabinose8-glucose, (2) luteo.6-glucose8-arabinose, (3) apig.6-rhaminose8-galactose, (4) naringin, (5) hespirdin, (6) rosmarinic acid

Data in Fig 1 shows the chromatographic flavonoids in aqueous extract of parsley, basil and chicory extract. Due to complexity of natural samples, identification of the every peak was impossible. The extract of parsley contain highest flavonoids content. The HPLC for the aqueous parsley extract showed presence of 16 compounds. The extract from parsley contained luteo.6-arabinose8-glucose, luteo.6-glucose8-arabinose, apig.6-rhaminose8-glucose, apig.6-glucose8-rhaminose, naringin, rutin, hespirdin, rosmarinic acid, apig.7-o-neohespiroside, apiginin-7-glucose, quercetrin, quercitin, naringenin, hespirtin, kampferol, apigenin with retention times of 9.41 min, 10.53 min, 11.78 min, 11.96 min, 12.17 min, 12.33 min, 12.37 min, 12.63 min, 12.85 min, 13.11 min, 13.26 min, 14.69 min, 14.92 min, 15.23 min, 15.94 min and 16.25 min respectively as appeared from the peaks areas (Fig. 1-a). The HPLC for the aqueous basil extract showed presence of 15 compounds. The extract from basil contained luteo.6-arabinose8-glucose, luteo.6-glucose8-arabinose, apig.6-rhaminose8-galactose, apig.6-rhaminose8-glucose, apig.6-glucose8-rhaminose, rutin, hespirdin, rosmarinic acid, apig.7-o-neohespiroside, apiginin-7-glucose, quercitin, naringenin, hespirtin, kampferol, apigenin with retention times of 9.39 min, 10.52 min, 11.45 min, 11.69 min, 12.01 min, 12.31 min, 12.36 min, 12.66 min, 12.75 min, 13.01 min, 14.67 min, 14.93 min, 15.25 min, 15.94 min and 16.26 min respectively as appeared from the peaks areas (Fig. 1-b). The HPLC for the aqueous chicory extract showed presence of 6 compounds. The extract from chicory contained luteo.6-arabinose8-glucose, luteo.6-glucose8-arabinose, apig.6-rhaminose8-galactose, naringin, hespirdin, rosmarinic acid with retention times of 9.42 min, 10.52 min, 11.42 min, 12.16 min, 12.35 min and 16.63 min respectively as appeared from the peaks areas (Fig. 1-c).

**Fig. 2:**
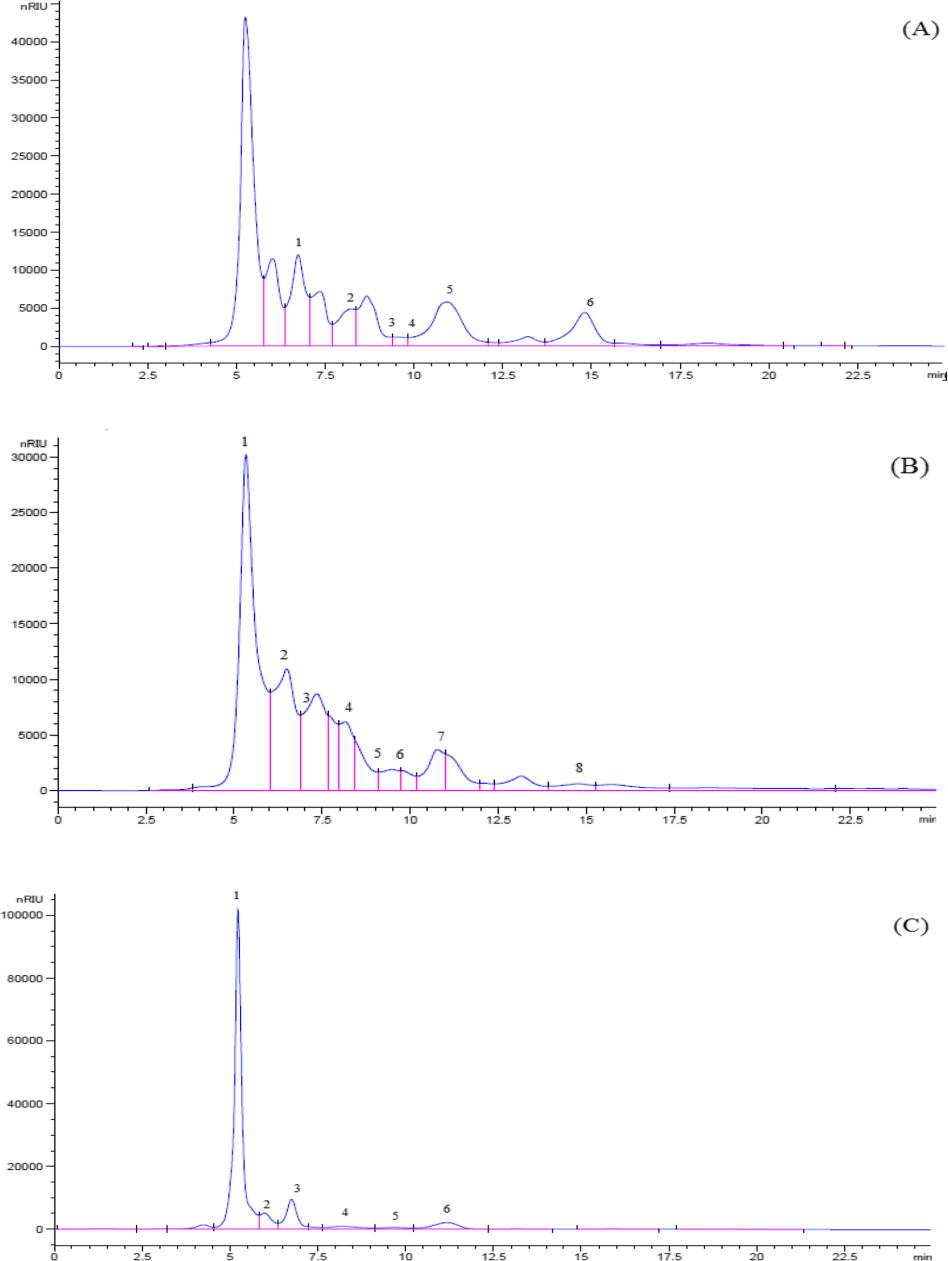
HPLC chromatogram of the saccharide compounds of: **a**: Petroselinum crispum leaf. The peaks were identified (1) sucrose, (2) glucose, (3) rhaminose, (4) mannose, (5) arabinose, (6) manitol. **b**: Ocimum basilicum leaf. The peaks were identified (1) glucuronic acid, (2) stachyose, (3) sucrose, (4) glucose, (5) xylose, (6) rhaminose, (7) arabinose, (8) manitol. **c**: Cichorium intybus L. leaf. The peaks were identified (1) inulin, (2) stachyose, (3) sucrose, (4) glucose, (5) mannose, (6) fructose

Data in Fig. 2 shows the chromatographic saccharides in aqueous extract of parsley, basil and chicory the extract. The extract of chicory contain inulin content. The HPLC for the aqueous parsley extract showed presence of 6 compounds. Their retention times are 6.74, 8.10, 9.43, 9.56, 11.92 and 14.8 which indicate the presence of sucrose, glucose, rhaminose, mannose, arabinose and manitol as appeared from the peaks areas (Fig. 2-a). The HPLC for the aqueous basil extract showed presence of 8 compounds. Their retention times are 5.337, 5.703, 6.767, 8.140, 9.162, 9.488, 10.984, 11.140 and 14.774 which indicate the presence of glucuronic, stachyose, sucrose, glucose, xylose, rhaminose, arabinose and manitol sorbitol as appeared from the peaks areas (Fig. 2-b). The HPLC for the aqueous chicory extract showed presence of 6 compounds. Their retention times are 5.230, 5.707, 6.755, 8.171, 9.665 and 11.173 which indicate the presence of inulin, stachyose, sucrose, glucose, mannose and fructose as appeared from the peaks areas (Fig. 2-c).

Data in Table 1 shows that the treatment of osteoporotic rats with different tested extracts produced a marked significant increase in concentration of estradiol, Ca, P and significant decrease in ALP and ACP activities in serum compared with their respective control. The treatment of osteoporotic rats with parsley and chicory extracts produced a marked significant decrease in the serum PTH and basil produced a non-significant decrease in PTH.

**Table 1:** Serum mineral parameters, enzyme activities in control and different treated groups

Groups	Normal	Dex.	Dex. + parsley	Dex. + basil	Dex. + chicory	P_ANOVA_	LSD at 5 %	LSD at 1 %
Parameters								
E2 (Pg/ml)	32.3 ± 6.45^a^	17.4 ± 0.86^c^	26.4 ± 2.31^b^	26.4 ± 0.38^b^	23.1 ± 0.73^b^	*P* < 0.001	3.699	5.006
PTH (Pg/ml)	8.36 ± 1.19^d^	17.4 ± 2.56^a^	14.0 ± 1.71^b^	15.6 ± 0.87^ab^	11.7 ± 0.91^c^	*P* < 0.001	1.882	2.546
Ca (mg/dl)	9.94 ± 1.14^a^	5.49 ± 0.38^c^	7.05 ± 0.63^b^	6.69 ± 0.83^b^	6.66 ± 0.62^b^	*P* < 0.001	0.908	1.228
P (mg/dl)	8.82 ± 0.39^a^	5.63 ± 0.46^c^	7.42 ± 1.07^b^	6.88 ± 0.69^b^	7.46 ± 0.81^b^	*P* < 0.001	0.851	1.152
ALP (IU/L)	199 ± 30.9^b^	393 ± 17.7^a^	202 ± 14.3^b^	203 ± 14.6^b^	210 ± 31.0^b^	*P* < 0.001	27.38	37.05
ACP (IU/L)	11.6 ± 2.16^d^	23.7 ± 1.62^a^	17.6 ± 2.93^b^	13.9 ± 1.62^cd^	16.5 ± 2.89^bc^	*P* < 0.001	2.758	3.731

Data are expressed as mean ± S.D. *N* = 6 animals per group

Mean with the different letters in the row are significantly different

*Dex.* dexamethasone, *E2* estradiol, *PTH* parathyroid hormone, *Ca* calcium, *P* phosphorus, *ALP* alkaline phosphatase, *ACP* acid phosphatase

In Table 2, treatment of osteoporotic rats with parsley, basil and chicory extracts showed significant increase in Ca and significant decrease in enzyme activities in bone when compared with their corresponding control. The treatment with basil and chicory produced significant increase in P bone concentration, while parsley induced a non-significant change when compared with osteoporotic group.

**Table 2:** Bone mineral parameters and bone enzyme activities in control and different treated groups

Groups	Normal	Dex.	Dex. + parsley	Dex. + basil	Dex. + chicory	P_ANOVA_	LSD at 5 %	LSD at 1 %
Parameters								
Ca (mg/g T)	15.9 ± 1.15^a^	10.2 ± 1.03^d^	15.5 ± 0.55^ab^	14.4 ± 0.93^b^	12.8 ± 0.75^c^	*P* < 0.001	1.081	1.463
P (mg/g T)	15.7 ± 0.49^a^	7.02 ± 0.16^d^	7.22 ± 0.47^d^	9.77 ± 0.91^c^	14.2 ± 0.72^b^	*P* < 0.001	0.716	0.969
ALP (IU/g T)	1186 ± 193^d^	2008 ± 89.2^a^	1396 ± 119^b^	1323 ± 94.9^cd^	1365 ± 142^b^	*P* < 0.001	157.9	213.6
ACP (IU/g T)	194 .1 ± 1.6^b^	213.1 ± 6.14^a^	197.6 ± 4.84^b^	195.9 ± 3.29^b^	195.1 ± 6.04^b^	*P* < 0.001	5.612	7.593

Data are expressed as mean ± S.D. *N* = 6 animals per group

Mean with the different letters in the row are significantly different

*Dex.* dexamethasone, *Ca* calcium, *P* phosphorus, *ALP* alkaline phosphatase, *ACP* acid phosphatase

Data presented in Table 3 proved that treatment of osteoporotic rats with parsley and basil extracts produced significant increase in total, proximal, mid BMD and BMC values, while chicory extract produced significant increase in proximal and mid BMD and a significant increase in total and proximal BMC when compared with the osteoporotic rats. Though, all extracts produced non-significant increase in distal BMD value compared with their control group.

**Table 3:** Bone mineral density and bone mineral contents in different areas in femur bone of control and different treated groups

Groups	Normal	Dex.	Dex. + parsley	Dex. + basil	Dex. + chicory	P_ANOVA_	LSD at 5 %	LSD at 1 %
Parameters								
Total BMD (mg/cm^2^)	60.5 ± 7.85^a^	38.7 ± 2.64^c^	50.9 ± 7.26^b^	51.5 ± 1.70^b^	55.5 ± 3.24^bc^	*P* < 0.001	6.176	8.355
Prox. BMD (mg/cm^2^)	55.8 ± 8.91^a^	43.9 ± 3.77^c^	52.2 ± 3.22^ab^	49.5 ± 2.12^bc^	52.7 ± 3.55^ab^	*P* < 0.001	5.853	7.919
Mid. BMD (mg/cm^2^)	69.0 ± 8.63^a^	38.2 ± 3.64^d^	61.7 ± 2.34^b^	54.7 ± 5.02^c^	54.9 ± 3.05^c^	*P* < 0.001	6.014	8.136
Dist. BMD (mg/cm^2^)	52.6 ± 6.63^a^	47.5 ± 6.61^a^	51.7 ± 4.79^a^	49.3 ± 3.86^a^	50.4 ± 4.13^a^	*P* > 0.05	-	-
Total BMC (mg)	83.0 ± 7.25^a^	28.5 ± 5.15^d^	50.4 ± 5.76^c^	60.8 ± 8.29^b^	55.0 ± 1.73^bc^	*P* < 0.001	7.271	9.837
Prox. BMC (mg)	43.6 ± 7.32^a^	12.4 ± 1.52^c^	27.3 ± 3.47^b^	24.5 ± 3.01^b^	25.0 ± 4.63^d^	*P* < 0.001	5.277	7.139
Mid. BMC (mg)	25.6 ± 4.17^a^	8.05 ± 1.33^d^	12.3 ± 1.56^c^	18.4 ± 3.19^b^	10.6 ± 0.78^cd^	*P* < 0.001	3.027	4.096
Dist. BMC (mg)	26.1 ± 2.87^a^	13.6 ± 2.49^c^	21.2 ± 1.03^b^	24.0 ± 2.47^a^	24.3 ± 0.87^a^	*P* < 0.001	2.541	3.401

Data are expressed as mean ± S.D. *N* = 6 animals per group

Mean with the different letters in the row are significantly different

*Dex.* dexamethasone, *BMD* bone mineral density, *BMC* bone mineral content, *Prox.* proximal, *Mid.* middle, *Dist.* distal

Changes in oxidative stress and antioxidant markers of bone are summarized in Table 4. Results showed that the treatment of osteoporotic rats with all examined extracts produced significant decrease of lipid peroxidation product while glutathione level was increased significantly as a result of chicory extract administration. Treatments with parsley, basil and chicory extracts induced significant increase of the glutathione-S-transferase and glutathione peroxidase activities as compared with the corresponding controls while the bone catalase activity was increased significantly as a result of basil and chicory extract administration only.

**Table 4:** Bone oxidative stress marker and antioxidant parameters in control and different treated groups

Groups	Normal	Dex.	Dex. + parsley	Dex. + basil	Dex. + chicory	P_ANOVA_	LSD at 5 %	LSD at 1 %
Parameters								
TBARS (nmol/g T)	18.1 ± 2.38^c^	56.3 ± 8.03^a^	32.1 ± 3.96^b^	30.4 ± 4.97^b^	23.5 ± 3.61^c^	*P* < 0.001	5.912	7.998
GSH (mg/g T)	6.76 ± 1.21^a^	2.37 ± 0.33^c^	2.96 ± 0.53^bc^	2.61 ± 0.54^bc^	3.49 ± 0.96^b^	*P* < 0.001	0.931	1.260
GST (U/g T)	2155 ± 254^a^	1277 ± 44.7^d^	1533 ± 59.2^c^	1648 ± 77.2^bc^	1741 ± 131^b^	*P* < 0.01	162.2	219.4
GPx (U/g T)	109 ± 21.7^a^	69.9 ± 7.58^b^	102 ± 11.5^a^	103 ± 16.5^a^	99.0 ± 13.8^a^	*P* < 0.01	18.08	24.46
GR (U/g T)	457 ± 67.9^a^	272 ± 70.7^b^	341 ± 88.6^b^	320 ± 55.9^b^	340 ± 91.2^b^	*P* < 0.01	90.40	122.3
Cat (U/g T)	0.97 ± 0.11^a^	0.32 ± 0.06^c^	0.37 ± 0.05^c^	0.59 ± 0.09^b^	0.88 ± 0.08^a^	*P* < 0.001	0.099	0.134

Data are expressed as mean ± S.D. *N* = 6 animals per group

Mean with the different letters in the row are significantly different

*Dex.* dexamethasone, *TBARS* thiobarbituric acid reactive substance, *GSH* glutathione reduced, *GST* glutathione s transferase, *GPx* glutathione peroxidase, *GR* glutathione reductase, *Cat* catalase.

### Histopathological

**Fig. 3:**
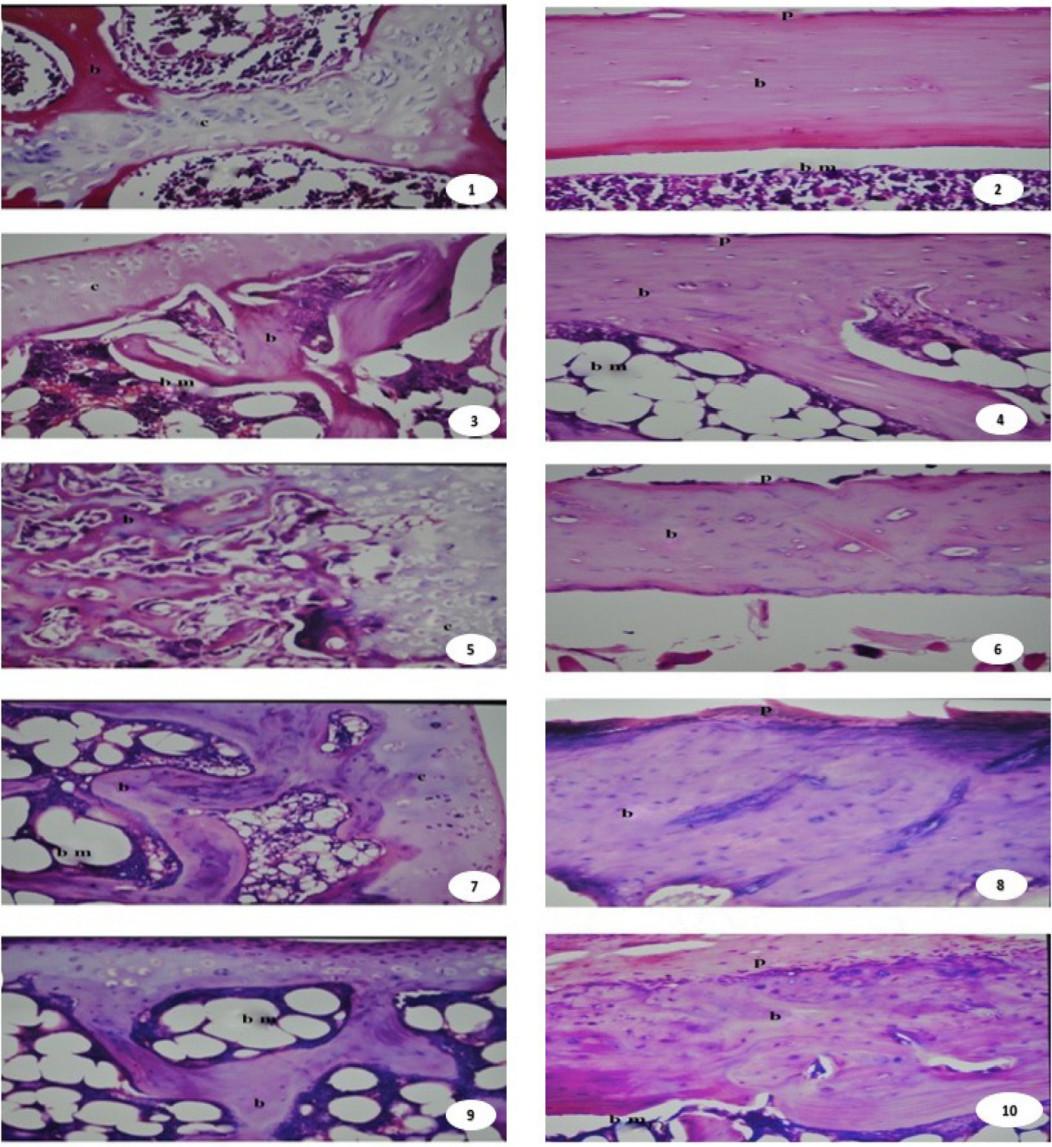
Photomicrographs of rat femur sections of different experimental groups stained with haematoxylin and eosin. (1 and 2): negative control group revealed no histopathological alteration in the condyle cartilaginous surface as well as the bony trabeculae and bone marrow. (3 and 4): the osteoporotic group revealed mild bone resorption in the trabeculae with intact Havarsian system. (5 and 6): Osteoporotic-treated rats with parsley extract showed bone resorption in the trabeculae associated with hypertrophy in the cartilaginous structure of the condyle and intact Haversian system in the shaft of the long bone. (7 and 8): rats treated with aqueous basil extract showed mild atrophy in the cartilaginous structure of the condyle with osteogenesis in the shaft. (9 and 10): rats treated with aqueous chicory extract revealed osteogenesis in both condyle and shaft of the long bone. X 100

From Fig. 3. Microscopically, the left femur of rat from negative control animals revealed no histopathological alteration in the condyle cartilaginous surface as well as the bony trabeculae and bone marrow (Photos 1& 2). Contrary, the osteoporotic group revealed mild bone resorption in the trabeculae with intact Havarsian system of long bone (photos 3& 4). Sections of parsley extract-treated rats showed bone resorption in the trabeculae associated with hypertrophy in the cartilaginous structure of the condyle and intact Haversian system in the shaft of the long bone (Photos 5& 6) while those of rats treated with aqueous basil extract showed mild atrophy in the cartilaginous structure of the condyle with osteogenesis in the shaft (Photos 7 & 8). Moreover, rats treated with aqueous chicory extract revealed osteogenesis in both condyle and shaft of the long bone (Photos 9 & 10).

## Discussion and conclusions

Dexamethasone decreased E2 levels associated with a significant decline in serum mineral concentrations, resulting in secondary hyperparathyroidism, which is consistent with the increase in PTH [37–39]. The enhanced bone turnover and fracture risk was reflected by the ALP activity [40]. Also, lack of inhibiting activity of estrogen on osteoclasts caused an increase in ACP activity and in consequence increase in bone resorption [41]. Glucocorticoids are known to alter the levels of TBARS and antioxidant enzymes in different tissue [42]. Increased free radicals production overwhelms the natural antioxidants defense mechanisms, subjecting individuals to hyperoxidant stress and thus leading to osteoporosis [43]. The decreased estrogen level in females increased the sensitivity of bones to the action of PTH leading to bone resorption with lower BMD [22] with concomitant decrease in bone matrix available for mineralization [44].

The ability of parsley extract to counteract the toxic effects may be attributed to the high nutritive value of parsley that concluded too high percent of vitamins (A, C, riboflavin and niacin) and minerals (Fe, Mg, P, K, Ca, Na and Zn). Essential trace elements are important parts of antioxidant enzymes as superoxide dismutase and glutathione peroxidase and may affect the antioxidant defense system [45]. Parsley contains vitamin k which positively affects calcium balance, a key mineral in bone metabolism and vitamin K insufficiency might be involved in the pathogenesis of osteoporosis [46–48]. The most potent osteogenic chemicals ever discovered as *petroselinum crispum* is quercetin and diosmetin glycosides [49, 50]. Quercetin induces apoptosis in mature osteoclasts and inhibits bone resorption and diosmetin induce osteoblastic differentiation [51, 52].

Administration of *O. basilicum* leaf extracts in dexamethasone treated rats tends to bring the bone MDA back to normal. Rosmarinic acid in *O. basilicum* suggested that it might have a role in the scavenging of free radicals [53]. Basil possessed good antioxidant properties attributed to free volatile aglycones in two different methods as the 2,2/-diphenyl-1-picrylhydrazyl radical scavenging method and ferric reducing/antioxidant power assay when compared with that of the essential oil and well known antioxidant butylatedhydroxytoluene [54]. Antiradical activity of phenolic compounds seen in *Ocimum* species depend on their molecular structure; and the availability of phenolic hydrogens, which result in the formation of phenoxyl radicals due to hydrogen donation [55]. An adequate supply of steroidal saponins of Anemarrhena asphodeloides prevented ovariectomized induced bone loss in rats through the promotion of bone formation [56]. Volatile oil of basil has estragol, linalool, eugenol, methyl chavicol and small quantities of methyl cinnamate, cineole, and other terpenes, apigenin, luteolin, orientin and vicenin [57], also, apigenin induced apoptosis of mature osteoclasts obtained from rabbit long bone and inhibited bone resorption [58]. The treatment with basil induced osteogenesis because *O. bacilicum* has a great number of compounds with oestrogenic activity [59] and phytoestrogens perform their antiosteoporotic effect by stimulating osteoblastic activity through an estrogen receptor mediated action, or by increasing the production of insulin 1 like growth factor-1 (IG-F) which is known to enhance osteoblastic activity [60].

In osteoporotic rats treated with chicory, it has been demonstrated that treatment with non-digestible fructans successfully increases Ca absorption and results in a corresponding increase in bone mineral [61] which is followed by a suppression of PTH, also non-digestible oligosaccharides from chicory roots on have the ability to reduce the elevation in the rate of bone turnover due to attributed to the ability to reduce the osteoclastic activity thus the rate of bone resorption decreases [38]. The phytochemical screening of chicory extract confirmed the presence of such bioactive compounds, particularly total phenolic, which may contribute to protection of chicory extract against free radical generation [62]. The treatment with chicory induced osteogenesis because *C. intybus L.* has osteoporosis preventive properties due to the protective effect of non-digestible oligosaccharides on bone in a rat model to mimic menopausal women was established through the following: 1) increased calcium absorption, 2) increased calcium balance, 3) increased bone mineralization, and 4) decreased bone turnover rate [63, 64].

### Conclusion

Bone mineral density, bone mineral contents raising effects as well as the anti-oxidant properties of chicory make it has more ability to prevented bone loss and decreased resorption of bone in the dexamethasone treated group. This suggests that chicory represents a promising therapeutic option for the prevention glucocorticoids- induced osteoporosis.

## Abbreviations

%, percentage; ACP, acid phosphatase; ALP, alkaline phosphatase; ANOVA, one-way analysis of variance; b, bony matrix; b. wt, body weight; bm, bone marrow; BMC, bone mineral content; BMD, Bone mineral density; C, Cartilage; *C. intybus L., Cichorium intybus L.*; Ca, Calcium; Dex., dexamethasone; DEXA, Dual energy x-ray absorptiometry; dist., Distal; E2, Estradiol; ELISA, Enzyme linked immunosorbent assay; Fe, Iron; GCs, Glucocorticoids; GIO, glucocorticoid-induced osteoporosis; gm, gram; GPx, glutathione peroxidase; GR, glutathione reductase; GSH, glutathione reduced form; GST, glutathione-S-transferase; H&E, hematoxylin and eosin; HPLC, high-performance liquid chromatography; IG-F, insulin 1 like growth factor; IU/g T, international unit per gram tissue; K, potassium; LSD, least significant differences; MDA, malondialdehyde; Mg, magnesium; Mid., middle; Na, sodium; *O. basilicum*, *Ocimum basilicum*; P, phosphorus; *P. crispum, Petroselinum crispum*; Pg/ml, picograms per milliliter; Prox., proximal; PTH, parathyroid hormone; r.p.m, round per minute; RI, refraction index; SD, standard deviation; TBARs, thiobarbituric acid reactive substances; UV, ultra violet; w/v, weigh/volume; Zn, zinc; μg, microgramme; μL, microliter.
